# Towards Deterministic Computation of Internal Stresses in Additively Manufactured Materials under Fatigue Loading: Part I

**DOI:** 10.3390/ma13102318

**Published:** 2020-05-18

**Authors:** Mustafa Awd, Mhd Fateh Labanie, Kerstin Moehring, Ali Fatemi, Frank Walther

**Affiliations:** 1 Department of Materials Test Engineering (WPT), TU Dortmund University, Baroper Str. 303, D-44227 Dortmund, Germany; labanie.mf@gmail.com (M.F.L.); kerstin.moehring@tu-dortmund.de (K.M.); frank.walther@tu-dortmund.de (F.W.); 2 Department of Mechanical Engineering, University of Memphis, Memphis, TN 38152, USA; afatemi@memphis.edu

**Keywords:** additive manufacturing, microcomputed tomography (µ-CT), fatigue loading, cyclic plasticity, Fatemi–Socie damage parameter

## Abstract

The ongoing studies of the influence of internal defects on fatigue strength of additively manufactured metals adopted an internal crack or notch-like model at which the threshold stress intensity factor is the driving mechanism of fatigue failure. The current article highlights a shortcoming of this approach and offers an alternative based on X-ray microcomputed tomography and cyclic plasticity with a hybrid formulation of Chaboche and Armstrong–Frederick material laws. The presented tessellation and geometrical transformation scheme enabled a significantly more realistic morphological representation of internal defects that yielded a cyclic strain within 2% of the experimental values. This means that cyclic plasticity models have an accurate prediction of mechanical properties without repeating a full set of experiments for additively manufactured arbitrary microstructures. The coupling with a material law that is oriented towards the treatment of cyclic hardening and softening enabled more accurate computation of internal stresses under cyclic loading than ever before owing to the maturity of tessellation and numerical tools since then. The resulting stress–strain distributions were used as input to the Fatemi–Socie damage model, based on which a successful calculation of fatigue lifetime became possible. Furthermore, acting stresses on the internal pores were shown to be more than 450% concerning the applied remote stress amplitude. The results are a pretext to a scale bridging numerical solution that accounts for the short crack formation stage based on microstructural damage.

## 1. Introduction

The free-form fabrication capability offered by additive manufacturing has left many structural and design issues inconclusively answered. Although titanium alloys have been in production by additive manufacturing for several years now [1,2], the reliable design capability of structural components of additively manufactured parts is still under question; which calls for the application of robust models [3,4]. The process-induced characteristics of technologies such as selective laser melting (SLM) and electron beam melting (EBM) result in quasi-static and fatigue strength; which are not similar to wrought Ti-6Al-4V alloy. Therefore; widely accepted design rules should be re-validated concerning additive manufacturing. Numerous research works have been dedicated to studying the relationship between process dynamics and structural properties on a meso- and microscale [5,6]

At this early phase of additive manufacturing application to Ti alloys, there were two main highlights of the research. The first is that micro-focused energy application on the feedstock imposes high thermal gradients on the solid solution that led to the formation of ultrafine-grained microstructures with extensive amounts of martensite. The second is that melt pool dynamics responsible for the formation of the keyhole and metallurgical pores under wide process windows become of significant interest of process researchers, as we see in a recent study combining experimental observation and numerical work [7]. One key highlight was that process technology is still facing challenges regarding stable energy delivery to the melt pool, especially in non-linear scan tracks, which are not unidirectional. Non-trivial scan tracks are unavoidable if service parts are to be produced by additive manufacturing. Attempts at solving the first and second issues in situ have been presented by [8,9] from two different points of view. The first focused on studying the decomposition of α′, which takes advantage of the ductility of the resulting α + *β*. The second has shown the influence of martensitic decomposition on cyclic fatigue properties, which have been induced by multi-exposure techniques during laser melting, which showed an improvement of ductility and fatigue strength as a result.

The study of fatigue strength of additively manufactured metals has focused on the point of view of the effects of defects, which has been supported by the findings of several researchers concerning the influence of process-induced defects on the failure mechanisms [6,10,11]. The interest in finding a correlation between internal defect size and resistance to fatigue crack initiation has been supported by the application of empirical models such as Kitagawa–Takahashi diagrams supported by defect size quantification methods such as fractography and X-ray microcomputed tomography (µ-CT) [12,13]. Recent research on defect analysis of defect distributions showed that, although 2D images can show the same trends in defect generation, 2D representation could not overtake the accuracy of 3D analysis in µ-CT proven by comprehensive statistical tests [14]. The application of the threshold principle to quantify the effect of internal defect treats it from the beginning as a notch or pre-crack, which disregards the influence of microstructure in slip band formation in the vicinity of the pore. The latter is widely accepted as a marginal phase of fatigue lifetime in the presence of internal defects, especially at critical locations. Instead of the threshold representation, other attempts utilized microcomputed tomography (µ-CT) to build three-dimensional finite element models that mimic the morphology of the internal pores, as has been progressively represented in the following research [15,16].

The current study aims at building on the concepts presented by this research with the introduction of two main developments. First is the application of a geometrical transformation road map of fatigue specimens from µ-CT to finite element models, which are more precise based on tessellation and decimation algorithms that have matured. The second is to present a tailored numerical tool based on a hybrid formulated model that accounts for hardening and softening under cyclic loading, which has its roots in the Armstrong–Frederick cyclic plasticity model [17]. The aim is to enhance the precision of the solution of the problem of computation of stress concentrations at internal defects and use the tool as a scale bridge for future analysis of microstructure–pore interaction at the microscale and robust modeling at the macroscale. The resulting stress–strain distributions are then used to calculate fatigue lifetime based on the maximum shear strain driven damage concept developed by Fatemi and Socie [18].

## 2. Materials and Methods

### 2.1. Experimental Methods

The model calibration and validation required the conduct of quasi-static and cyclic tests on selective laser melted (SLM) AlSi10Mg and Ti-6Al-4V. All specimens were melted in an SLM Solutions 250 HL with laser power of 250 W and 1000 W for AlSi10Mg and Ti-6Al-4V, respectively. The perpendicular direction to the building platform was maintained for all specimens used in the study. Round bars, which were vertically manufactured, were machined and polished to the final specimen geometry as the influence of surface roughness is excluded from this study. A second aim is to study only the deeply generated defects inside the bulk of the specimens, which are different in morphology and formation mechanism to the subsurface defects in the skin of the as-built structures. Hence, the effect of surface defects and their interaction with the notches of surface roughness is postponed to a further study, which should isolate this effect. For further details on the manufacturing process, heat and surface treatments, as well as scanning parameters, the reader can be referred to the articles of [9,19].

The quasi-static tensile tests were conducted on an Instron 3369 system with a 50 kN load cell, where force–displacement curves were recorded and used to obtain stress–strain curves. For AlSi10Mg, the cyclic load increase test (LIT) was applied at an Instron 8872 system with a 10 kN load cell. A testing program, which starts at a load equivalent to 30 MPa of stress amplitude (well below any observable cyclic plastic strain by the available measurement techniques’ precision), is continuously increased at a rate of 10 MPa/10^4^ cycles. Corresponding strain characteristics were recorded for the test. On the other hand, Ti-6Al-4V specimens were tested at Schenck PC63M/Instron 8800 system with a load capacity of 45 kN, such that the starting stress amplitude was 150 MPa and load increment slope was set to 20 MPa/10^3^ cycles. Constant amplitude tests (CATs) were conducted for validation at the respective systems and load levels based on the results of the load increase tests.

Specimens were scanned before testing at Nikon X TH 160 equipped with a 160 kV gun and an amorphous silicon-based digital detector with a 1024 × 1024-pixel count that corresponds to 127 μm pixel pitch. Further details on scanning parameters can be found in [20]. For precise capturing of pore morphology, a calibration process was performed before scanning to eliminate shading errors. On VGStudio max 2.2, the raw data of microcomputed tomography (µ-CT) were processed in order to visualize the three-dimensional defect distribution inside the specimen. Furthermore, using the same software, surfaces of the specimens and internal surfaces of the pores were extracted and consequently processed in a Python-based CAD software, where pore surfaces about the bulk specimen were stitched and joined. Unfortunately, the maximum resolution of the µ-CT scan (minimum equivalent diameter detected = 20 µm) could not be transferred to a three-dimensional model, as this would have resulted in a tessellation containing >2.5E6 vertices. Therefore, the model underwent several simplifications and reduction filters to be feasible to study by the finite element method. On VGStudio max 2.2, a point reduction was conducted based on a tolerance range. If a vertex was outside the tolerance range from the neighboring vertices, it was deleted. The rest of the points were preserved to save the structure topography. The second filter was conducted on MeshLab using a quadric edge-collapse decimation algorithm, where neighboring tetrahedral elements forming a single surface were joined to form a single element. Although these simplification processes compromised the quality of geometrical representation to some extent, the remedy applied by the investigators was to conduct a proper mesh sensitivity analysis, as will be shown in a following section.

Sections of AlSi10Mg and Ti-6Al-4V were hot-embedded and then ground and polished by grit paper from 320 down to 4000 grade grinding paper with water lubrication. Polishing was done using a 3–1 µm diamond suspension, which was followed by active polishing using an Oxide polishing suspension (OPS) suspension. The electron backscatter diffraction (EBSD) was conducted on a scanning electron microscope (SEM) of Tescan Mira XMU. Ti-6Al-4V specimens required more parametric resolution owing to significantly finer lamella. The EBSD maps were filtered and analyzed to produce three-dimensional models of the microstructure based on several EBSD maps of the same specimen. The microstructural models, which will also be used in a specific following study, are used here to establish a scale-bridge to correlate macroscale properties to microscale morphology concerning internal stresses on the mesoscale, which are calculated by cyclic plasticity.

### 2.2. Theory and Calculations

The cyclic deformation simulation and fatigue lifetime calculation algorithm begin with a material parameter identification scheme to satisfy the hardening rules, which were intended to be applied in a combined manner [21,22]. The classical parameter identification methodologies described by [23,24,25] are costly in terms of their effort and economy of the experiments themselves. Here, we apply a parameter identification method, which is an extension of the work of [9], which relies on a quasi-static tensile test as well as the continuous load increase test. The quasi-static test is applied to calibrate isotropic hardening parameters, Q. The load increase tests calibrate the kinematic hardening parameters k, C, and γ with respect to the requested number of back stresses necessary to capture hysteresis translation in the three-dimensional space. The proposed technique allows identification of isotropic and kinematic hardening of everyone on its own without interaction, as two independent tests are used. An incremental increase of stress is applied on Abaqus, where stress amplitude levels are predefined and compared to the measured plastic strains from the load increase test, so parameters of the following kinematic hardening rule can be identified [21]:

(1) $$\frac{\Delta \sigma }{2}-k=\frac{C}{\gamma }\tanh\left(\gamma \frac{\Delta \varepsilon^{pl}}{2}\right)$$

where C and γ are material parameters related to the evolution of kinematic hardening law, and k is the initial yield stress. The Armstrong–Frederick model requires the identification of one back stress. In a general cyclic loading test, the yielding condition will be expressed as follows [25]:

(2) F=(32(σ′−x′):(σ′−x′))12−σy(p)

(3) $$\sigma_{y}\left(p\right)=\sigma_{y0}+\sigma \left(p\right)$$

where $\sigma^{\prime }$ is a deviatoric tensor of the stress component, $x^{\prime }$ is the deviatoric tensor of the back stress, $\sigma_{y}\left(p\right)$ is the yield stress as a function of accumulated plastic strain, $\sigma_{y0}$ is the initial yield stress, and σ(p) is the increment in the yield stress as a function of accumulated plastic strain. The accumulation of plastic strain in the three-dimensional space can be expressed as follows:

(4) dp=p˙=(23ε˙pl:ε˙pl)12≈(23ε˙:ε˙)12

Under the presented approximation above, it can be stated that plastic damage in the uniaxial fatigue can be set equal to the rate of strain in the same direction. Similarly, the deviatoric part of the back stress is simplified, and volume constancy principle is accounted for, which yields the following simplification in the current case of uniaxial fatigue loading:

(5) F=(3223(σ11−x11)2)12−σy(p)=|σ11−x11|−σy(dε11)

for which a formulation of a specific flow rule for the current aim considers isotropic and kinematic hardening, which translates the yield surface in space based on the load memory that can be divided into infinitesimal steps. The utilized incremental plastic strain is formulated as follows:

(6) $$d\varepsilon^{pl}=d\lambda \;r\left(\sigma ,x,\;p\right)$$

where dλ is a consistency parameter, describing the magnitude of evolution or translation of the yield surface, while r(σ,x, p) is a function that describes the direction of evolution or translation. Here, a Drucker stability postulate is considered as well as the yielding principle of von Mises. For metals, an associated flow rule that is utilized as a direct function of stress is given by the following:

(7) $$d\varepsilon^{pl}=d\lambda \;sgn\left(\sigma -x\right)$$

A simplification of the hardening rules of isotropic and kinematic hardening associated with Armstrong–Frederick is applied to the case of unidirectional loading [22]. A multiplier ψ is used, which yields the upcoming hardening rule:

(8) $$dx=Cd\varepsilon^{pl}-\gamma x\left|d\varepsilon^{pl}\right|=Cd\varepsilon^{pl}-\gamma x\psi d\varepsilon^{pl}=\left(C-\gamma x\psi \right)d\varepsilon^{pl}$$

The elastic-plastic model is numerically solved in an implicit integration scheme based on a Newton–Raphson algorithm according to [26], where it was implemented in cyclic deformation simulation in Abaqus using a UMAT subroutine and a consistent tangent operator according to [27] for rate-independent elastoplasticity.

**Figure 1:**
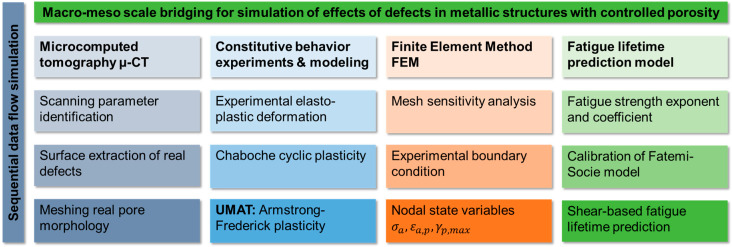
Flow chart of the applied simulation scheme on the macro-mesoscale.

The resulting stresses during the fatigue simulations at internal porosity were mainly multiaxial in a very localized plastic deformation fashion, which, on the micro-nanoscale, are the driving force for slipping of persistent shear bands. The latter is closely aligned with the maximum shear strain direction according to [18], which makes fatigue crack initiation increasingly associated with the maximum shear plane. The relationship between shear strain based on the Brown–Miller equation [28] and normal stress, formulated by Fatemi and Socie in [18], is given by the following:

(9) $$\gamma_{max}\left(1+k\frac{\sigma_{n}^{max}}{\sigma_{y}}\right)=constant$$

where $\gamma_{max}$ is the maximum shear strain, and k is the Fatemi–Socie damage parameter calibrated by fitting the uniaxial and torsional stress data. The fatigue lifetime can be predicted based on the maximum shear strain as well as maximum normal stress under consideration of material hardening, nucleation, and growth of small cracks, as suggested by the following [29]:

(10) $$\frac{\Delta \gamma_{max}}{2}\left(1+k\frac{\sigma_{n}^{max}}{\sigma_{y}}\right)=\gamma_{f}^{\prime }{\left(2N_{f}\right)}^{c_{0}}+\frac{\tau_{f}^{\prime }}{G}{\left(2N_{f}\right)}^{b_{0}}$$

where $\Delta \gamma_{max}$ will be calculated during the cyclic deformation simulation in Abaqus. Solving Equation (10) for $2N_{f}$ The fatigue lifetime in cycles of an actual specimen can be predicted based on the precision of the geometrical representation of internal porosity and numerical accuracy of the implementation of the Armstrong–Frederick model. An overview of the whole process from the initial experiments until the calculation of fatigue lifetime is given in Figure 1. The suggested methodology begins with performing µ-CT scans of the fatigue specimens. The surfaces of the specimens are extracted and transformed into solid meshed models. The formulated plasticity model is calibrated based on quasi-static and cyclic tensile tests and implemented in a UMAT code in Abaqus. The finite element (FE) simulation involves performing a sensitivity mesh analysis and setting boundary conditions that mimic the experimental conditions. In the lifetime prediction step, the Fatemi–Socie damage model is calibrated, and maximum shear strains developed in the simulation are fed into the model to calculate the lifetime.

## 3. Results and Discussion

### 3.1. Experimental Results

**Figure 2:**
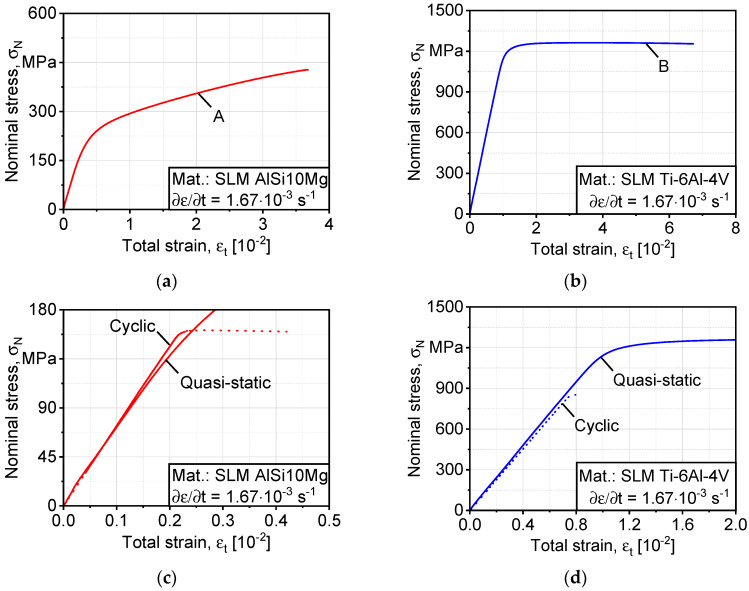
Quasi-static stress–strain curves used to deduce isotropic hardening parameters: (**a**) AlSi10Mg; (**b**) Ti-6Al-4V, in the applied cyclic load range; (**c**,**d**) comparison between cyclic hardening (AlSi10Mg) and softening (Ti-6Al-4V) in both alloys. SLM, selective laser melted.

Quasi-static tests were executed at a displacement rate of 1 mm/min, and force–displacement curves were recorded in addition to the application of an extensometer, which had a 10 mm gauge length. Figure 2 shows the stress–strain curves that resulted based on the tensile tests for SLM AlSi10Mg and Ti-6Al-4V. The curves were used to calibrate isotropic hardening parameters b, Q based on virtual expansions of yield surface in Abaqus, as explained in Section 2.2. The elastic moduli are ~70 GPa and ~120 GPa, respectively, on an average of three tests. Therefore, the actual value was used in the simulation, as well as the actual flow curves. On the other hand, the hardening modulus of AlSi10Mg is significantly higher, as seen in the curves.

**Figure 3:**
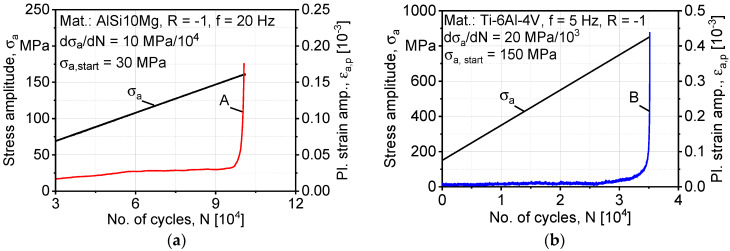
Cyclic stress–plastic strain amplitude curves used to deduce kinematic hardening parameters: (**a**) AlSi10Mg; (**b**) Ti-6Al-4V.

Figure 3 shows the cyclic completely reversed (stress ratio R = −1) load increase testing curves, which were used to calibrate the kinematic hardening parameters k, C, and γ in Equation (1). It is worth mentioning that AlSi10Mg shows more active cyclic softening behavior than Ti-6Al-4V as load increases, which underlies the most significant difference in the structure morphology as AlSi10Mg is strengthened by fine interdendritic eutectic Si arms, while Ti-6Al-4V relies mainly on martensitic *α′* with fine lamella for the strength of the microstructure [2,20].

**Figure 4:**
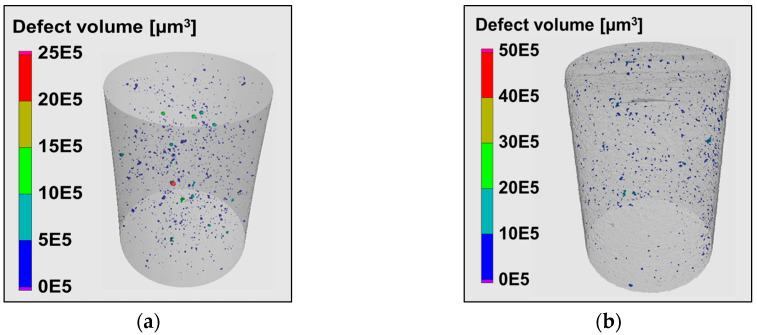
Gauge length scans and 3D defect distributions used in the cyclic deformation simulations: (**a**) AlSi10Mg; (**b**) Ti-6Al-4V.

The specimens of the study, which were produced by optimized scanning parameters, had a relative density well above 99.50% [9,19]. Nevertheless, a single specimen contained several thousand micropores, as revealed by the µ-CT analysis. The 3D defects distributions are shown in Figure 4, where the geometrical transformation was handled in STL shell representation. A Boolean differential operator was run in a Python script to extract pores with the exact morphology from the computed tomography, which resulted in a single solid specimen geometry with internal defects imitating the pores resulting from the selective laser melting process.

**Figure 5:**
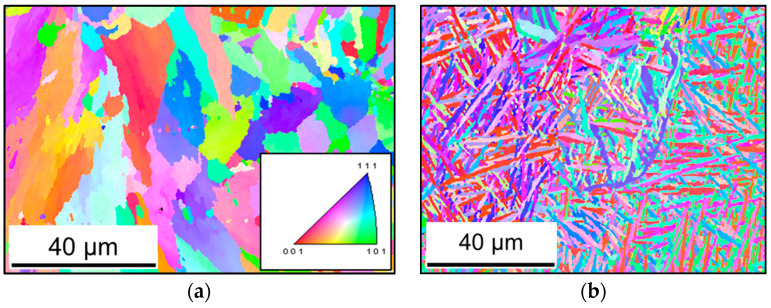
Texture analysis of investigated alloys: (**a**) AlSi10Mg; (**b**) Ti-6Al-4V.

Crystallographic anisotropy is another structural discontinuity that has an influence on the crack initiation process at the local maximum shear strain planes of internal pores. In Figure 5, a significant texture is observed for Ti-6Al-4V, where the crystals are finer with a lamellar-weave morphology. The gliding of dislocations and grain boundaries under cyclic loading is thus resisted, as in Figure 3. Recent literature has concluded that the anisotropy and heterogeneity in the mechanical properties of metal additively manufactured parts are because of microstructural texture and anisotropy [30]. The treatment of microstructure of Ti-6Al-4V by hot isostatic pressing (HIP) led to significant differences in fracture toughness and crack growth resistance under fatigue loading [31].

### 3.2. Modeling and Simulation

**Figure 6:**
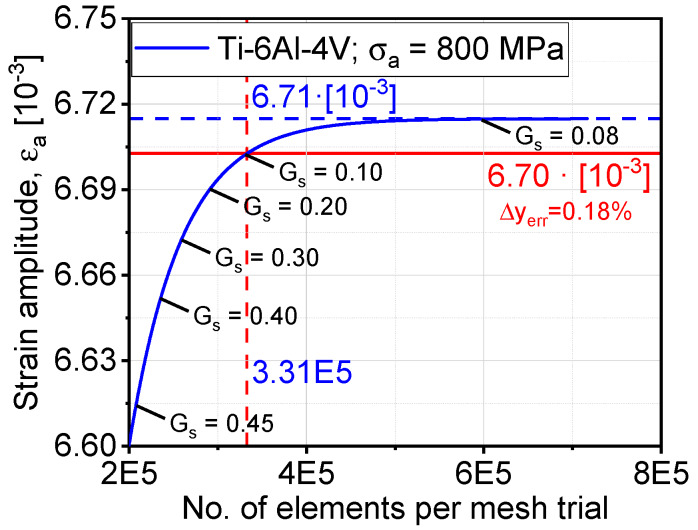
Mesh sensitivity analysis for the determination of an optimum number of elements and element size.

The mesh sensitivity analysis was performed on a Ti-6Al-4V model of a fatigue specimen under a stress amplitude of 800 MPa at a stress ratio of R = −1. The convergence criteria adopted here are based on the maximum developed strain after reaching the stabilized cyclic response in the simulation. Hence, a reasonably accurate, yet efficient FE model can be built; therefore, we assign a global seed ($G_{s}$) distribution with which the number of nodes and elements are initialized. Six different levels of $G_{s}$ distributions are selected, as shown in Figure 6, where the number of elements is plotted against the strain amplitude. By decreasing $G_{s}$, the spatial distance between two vertices decreases. For further simulations, $G_{s}$ was set to 0.10, where it yielded a precision of ~99.80% for the saturated value at $G_{s}$ = 0.08. The simulations were performed under force control for fatigue lifetime prediction. The highlight of mesh sensitivity analysis is to compensate for the numerical accuracy compromised by the decimation algorithm of Section 2.1.

**Figure 7:**
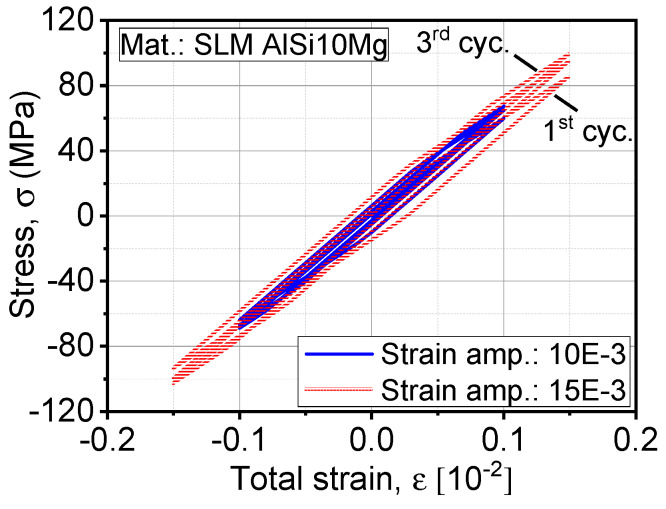
Expansion of hysteresis under the influence of increased strain as solved by the Armstrong–Frederick model.

The comparison between continuous hysteresis loops, like the one in Figure 7, exhibits a reduction in the plastic strain energy density (i.e., area bounded by hysteresis loop). The plastic damage tolerance of an alloy is the ability of the materials to withstand more load reversals, which affects the material’s fatigue limit. After saturation is reached, plastic damage continues to accumulate, which is a result of yield surface translation in the three-dimensional stress field. In Figure 7, we observe a cyclic hardening, which is represented by reaching higher stresses at two controlled levels of displacements. It agrees well with the experimental observation in Figure 2c. On the other hand, Ti-6Al-4V showed a cyclic softening behavior. At this point, not only isotropic hardening is contributing to the plastic damage process; on the other hand, kinematic hardening is the primary driving mechanism of plastic damage as it shifts the hysteresis from the origin in the stress space. This phenomenon is more pronounced in the case of asymmetrical loading R ≠ 1. The predicted strains by the FE model were within 1% of the experimental value at 800 MPa for Ti-6Al-4V and 2% at 600 MPa of stress amplitude. On the other hand, the model calibration was based on the tensile quasi-static and cyclic load increase tests, which were used to predict the behavior under constant amplitude testing.

Simulations that were done on meshes of computed tomography defect volumes delivered results of stress–strain distribution all over the fatigue specimen, including damage distributions around internal pores. Simulations were run until cyclic saturation depending on a Newton–Raphson integration algorithm, which does not call for the simulation of single cycles using the following Fourier series [32]:

(11) $$\bar{u}\left(t\right)=u_{o}+\overset{n}{\underset{k=1}{\sum }}\left[u_{k}^{s}\;sin\left(k\omega t\right)+u_{k}^{c}\;cos\left(k\omega t\right)\right]$$

(12) $$\bar{R}\left(t\right)=R_{o}+\overset{n}{\underset{k=1}{\sum }}\left[R_{k}^{s}\;sin\left(kwt\right)+R_{k}^{c}\;cos\left(kwt\right)\right]$$

where n is the no. of Fourier terms; ω=2π / T is the angular frequency; and $R_{o}$, $R_{k}^{s}$, and $R_{k}^{c}$ correspond to displacement coefficients $u_{o}$, $u_{k}^{s}$, and $u_{k}^{c}$ respectively.

**Figure 8:**
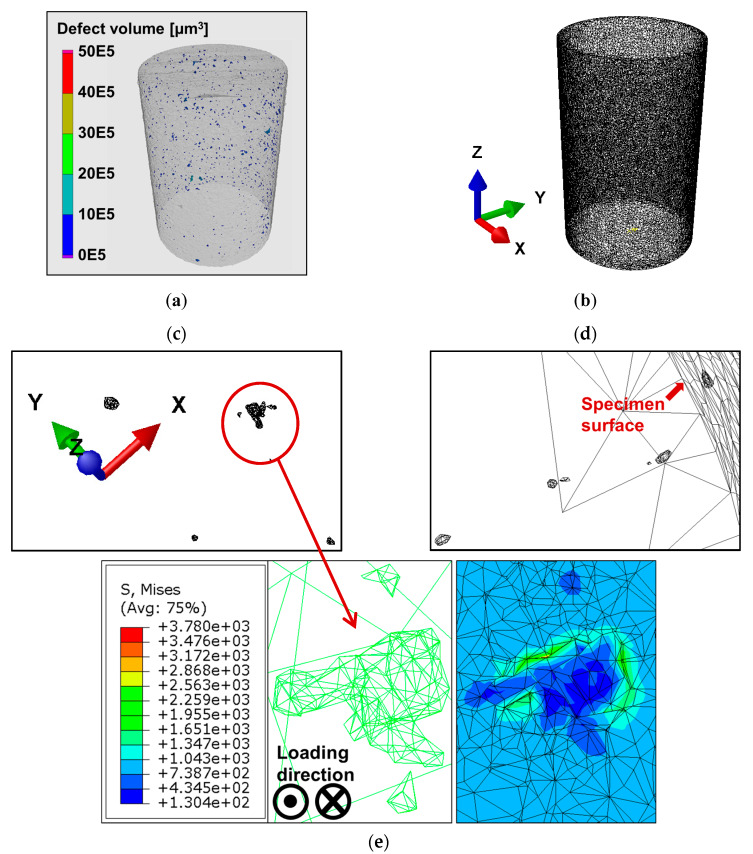
Process chain of porosity analysis from microcomputed tomography (µ-CT) scan until stress calculation around internal remnant pores when the applied remote stress amplitude is 800 MPa: (**a**) 3D defect volume distribution; (**b**) 3D surface tessellation of the scanned volume; (**c**) clusters of defects concerning the coordinate system in the bulk of the scanned volume; (**d**) multiple defects at different depth levels from the surface (surface is at the top right) respecting the same triad in (**c**); (**e**) stress concentration at a cluster of non-spherical defects.

**Figure 9:**
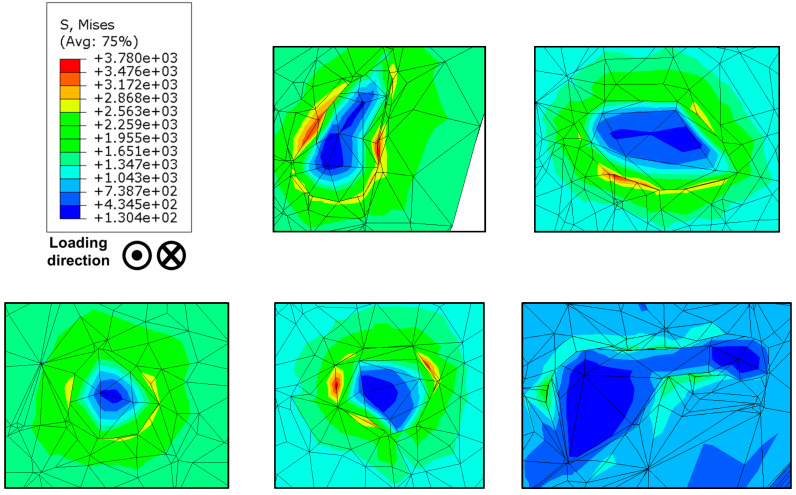
Stress distribution at multiple pores inside Ti-6Al-4V specimen cyclically loaded at stress ratio R = −1 and stress amplitude $\sigma_{a}$ = 800 MPa.

In Figure 8, the process is represented stepwise, where a µ-CT scan of a Ti-6Al-4V specimen is transferred to a finite element mesh according to Section 2.1. In Figure 8c, a cluster of subsurface pores is magnified, where we see a shrinkage pore typical of SLM and cast alloys owing to the collapse of gas pressure upon cooling. In Figure 8e, the mesh is shown with $G_{s}$ = 0.1. In addition to the primary defects, two smaller pores at twelve and six o’clock directions exist, which represent a likely location of crack coalescence under cyclic loading. Furthermore, the stress distribution around the defect is shown under a cyclic stress amplitude of 800 MPa; on the other hand, the internal stress on the defects is more than 450% to the applied remote stress amplitude. The potential directions of crack progression from this defect are highlighted by the stress distribution legend. The influence of microstructural damage at these locations is critical to the early crack formation phase. To capture such small microstructural features and still get a feasible converging FE model, a representative volume element is proposed later in the discussion. In Figure 9, further pores with as-built random-like morphology are shown with their corresponding stress distributions.

**Figure 10:**
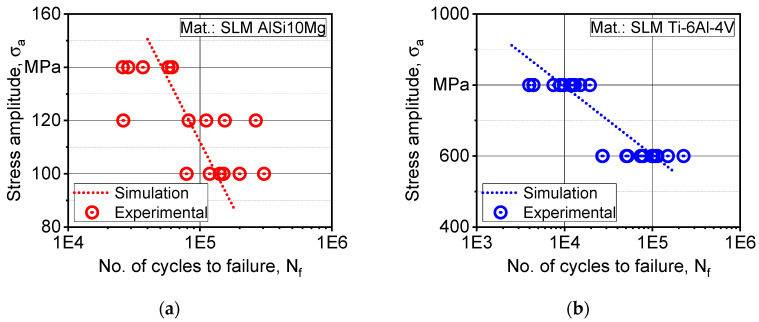
Evaluation of the proposed modeling scheme by comparison with experimental fatigue data: (**a**) AlSi10Mg; (**b**) Ti-6Al-4V.

The fatigue lifetime prediction model based on the Fatemi–Socie model in Equation (10) depends on the maximum in-plane shear strain developed under simulated cyclic loading. The damage parameter k was determined in an inverse iterative manner based on simulation data of von Mises shear strain amplitude. In Figure 10, we see a comparison of numerical and experimental fatigue data, where a good fit is found in the load range where this simulation is applied.

On the other hand, the authors question if the same degree of accuracy can be found in late high-cycle and very high-cycle fatigue (VHCF), where the convergence of the used plasticity model is not precise owing to low plastic strain increments. Therefore, the need for material laws at a lower scale is foreseen. The crystal plasticity constitutive laws are recognized as valid laws at the scale of the microstructure around pores presented in Figure 8. By application of crystal slipping models [33], converging damage parameters at low remote stresses typical of very high-cycle fatigue (VHCF) can be obtained:

(13) $${\hat{L}}^{p}=\overset{N_{sys}}{\underset{\alpha =1}{\sum }}{\dot{\gamma }}^{\alpha }\left({\bar{s}}^{\alpha }⨂{\bar{n}}^{\alpha }\right)={\dot{F}}^{p}F^{p^{-1}}$$

where ${\dot{\gamma }}^{\alpha }$ is the slip system shearing rate and ${\bar{s}}^{\alpha }$ and ${\bar{n}}^{\alpha }$ are unit vectors in the slip direction and slip normal plane direction, respectively, for the $\alpha^{th}$ slip system. The viscoplastic flow rule for the shearing rate on the $\alpha^{th}$ slip system is given by:

(14) $${\dot{\gamma }}^{\alpha }={\dot{\gamma }}_{0}{\langle \frac{\left|\tau^{\alpha }-x^{\alpha }\right|-{Κ}^{\alpha }}{D^{\alpha }}\rangle }^{m}sgn\;\left(\tau^{\alpha }-x^{\alpha }\right)$$

where ${\dot{\gamma }}^{\alpha }$ is the reference shear rate, $\tau^{\alpha }$ is the resolved shear stress, $\chi^{\alpha }$ is the back stress, ${Κ}^{\alpha }$ is the length scale-dependent threshold stress, $D^{\alpha }$ is the drag stress, and m is the flow exponent. The crystal slipping constitutive laws are widely applied to statistical and representative volume elements to obtain crack initiation lifetimes [34,35]. On this scale [35], attractive contributions were made based on the segmentation of single grains in the microstructure and were able to calculate crack initiation lifetime based on Tanaka–Mura dislocation gliding theory. Such micromechanical simulations were shown to be effective at enhancing the precision of model calibration on the macro scale, as shown by [36]. Fatemi et al. highlighted that unforced fatigue crack that will initiate from slip bands on the maximum shear planes would adopt a mode-II shearing failure that is likely to branch into mode-I tensile failure depending on the non-homogeneities the crack will encounter in its path [37,38]. The phenomenon was visualized in interrupted very high-cycle fatigue (VHCF) fatigue tests by Awd et al. in [20] in SLM AlSi10Mg, where shear occurring cracks branched and developed into further modes depending on local microstructure and porosity as well local loading variations at various phases in a specimen’s life.

Additive manufacturing is always praised for the capability of producing components of high complexity, which, under service loading, will develop complex multiaxial stresses. Although fatigue data presented in this study and their analysis are uniaxial, all main elements necessary to conduct a multiaxial stress analysis of durability are present in the analytical approach discussed here. The combined application of 3D µ-CT data, FE model, cyclic plasticity material law, as well as Fatemi–Socie damage parameter, is utilized here for the advantageous handling of complex AM geometries that are likely to experience multiaxial stress states. A focused investigation of this statement will follow in a separate study.

## 4. Summary

The preliminary investigations for this study realized a tendency in the literature to model the influence of defects in additively manufactured materials as precracks where the threshold stress intensity factor is the main damage parameter indicator. Although the approach is supported by failure analysis investigation, which saw fatigue fracture initializing at critical internal defects for polished specimen, the approach overlooks the influence of microstructural resistance at notch and pore roots, which will have a significant influence in very high-cycle fatigue regime with a substantial fraction of fatigue lifetime.

This point encouraged authors to develop a previously introduced concept to evaluate the effects of defects, studying the actual acting stresses on pores utilizing microcomputed tomography and the finite element method. Here, the methodology to transfer X-ray scans to finite element models was enhanced by filtered tessellation of surfaces, Boolean operators, and vertex decimation algorithm. To economize on the number of experimental tests without compromising precision, a calibration procedure was presented based on a cyclic load increase test for isotropic and kinematic hardening parameters as well as virtual expansions of yield surfaces.

The rate-independent cyclic plasticity law of Armstrong–Frederick is hybridlike formulated with kinematic hardening parameters from Chaboche law with single back stress. The formulation is simplified for a case of uniaxial cyclic loading in order to focus on the cyclic hardening phenomena and compare it for two different alloys. The formulation with an associative plastic flow rule was found to converge for sufficiently large increments and stress levels. The resulting stress–strain distributions were used with the Fatemi–Socie equation to calculate fatigue lifetimes based on maximum shear strain, which was sufficiently successful in a certain load range for AlSi10Mg and Ti-6Al-4V. The corresponding hardening characteristic of the presented constitutive formulation was shown within the experimental strain range for a sufficiently large number of elements.

The simulations of fatigue damage based on macroscale back-stress hardening revealed a limitation of convergence and real damage representation at low stresses typical of very high-cycle fatigue, where low increments hindered convergence of the tangent modulus. Besides, the resulting stress distributions at pore roots highlighted the potential of analyzing microstructural damage at these locations in order to calculate fatigue lifetime on two integrating scales. The first one is the short crack formation time added to the second one, which is long fatigue crack propagation, which fully represents a fatigue lifetime of a service component. To this end, various elements of the current study were assembled in discussion with concepts from the available literature about the use of three-dimensional representative volume elements, coupled with crystal plasticity finite element modeling that forms an input to a crystal-based crack initiation criterion.
